# Long-Term Outcomes of a Phase I Study With UV1, a Second Generation Telomerase Based Vaccine, in Patients With Advanced Non-Small Cell Lung Cancer

**DOI:** 10.3389/fimmu.2020.572172

**Published:** 2020-11-26

**Authors:** Paal F. Brunsvig, Tormod Kyrre Guren, Marta Nyakas, Claudius H. Steinfeldt-Reisse, Wenche Rasch, Jon Amund Kyte, Hedvig Vidarsdotter Juul, Steinar Aamdal, Gustav Gaudernack, Else Marit Inderberg

**Affiliations:** ^1^ Department of Clinical Cancer Research, Oslo University Hospital–The Norwegian Radium Hospital, Oslo, Norway; ^2^ Department of Radiology and Nuclear Medicine, Oslo University Hospital, Oslo, Norway; ^3^ Ultimovacs ASA, Oslo, Norway; ^4^ Department of Cellular Therapy, Oslo University Hospital–The Norwegian Radium Hospital, Oslo, Norway

**Keywords:** non-small cell lung cancer, human telomerase reverse transcriptase, peptide, vaccine monotherapy, dose response, clinical efficacy

## Abstract

Human telomerase reverse transcriptase (hTERT) is a target antigen for cancer immunotherapy in patients with non-small cell lung cancer (NSCLC). We have tested a novel hTERT vaccine, UV1, designed to give high population coverage. UV1 is composed of three synthetic long peptides containing multiple epitopes identified by epitope spreading data from long-term survivors from previous hTERT vaccination trials. Eighteen non-HLA-typed patients with stage III/IV NSCLC with no evidence of progression after prior treatments, were enrolled in a phase I dose-escalation study of UV1 vaccination with GM-CSF as adjuvant, evaluating safety, immune response, and long-term clinical outcome. Treatment with UV1 was well tolerated with no serious adverse events observed. Seventeen patients were evaluable for tumor response; 15 patients had stable disease as best response. The median progression free survival (PFS) was 10.7 months, and the median overall survival (OS) was 28.2 months. The OS at 4 years was 39% (7/18). Five patients are alive (median survival 5.6 years), and none of these are known to have received checkpoint therapy after vaccination. UV1 induced specific T-cell responses in the majority (67%) of patients. Immune responses were dynamic and long lasting. Both immune response (IR) and OS were dose related. More patients in the highest UV1 dosage group (700 μg) developed IRs compared to the other groups, and the IRs were stronger and occurred earlier. Patients in this group had a 4-year OS of 83%. The safety and clinical outcome data favor 700 μg as the preferred UV1 dose in this patient population. These results provide a rationale for further clinical studies in NSCLC with UV1 vaccination in combination with immune checkpoint blockade.

**Clinical Trial Registration:**

https://www.clinicaltrials.gov, identifier NCT0178909.

## Introduction

Lung cancer is among the most common types of cancer and the leading cause of cancer death in men and women worldwide, with non-small cell lung cancer (NSCLC) accounting for over 85% of all cases. At the time of diagnosis, the majority of cases are at a locally advanced stage (stage III) or with metastases (stage IV) (1). For several decades, treatment of patients with stage III or IV NSCLC has been chemotherapy and/or radiotherapy often with a limited improvement in survival.

During the last few years immunotherapy, i.e., anti-PD-1/PD-L1 antibodies, has emerged as the most promising treatment option for NSCLC patients. The widespread use of immunotherapy in patients with NSCLC has come exceptionally quickly, starting from the first report of objective response to PD-1 blockade in 2012, to the first FDA approval in 2015 (2). About 20–25% of NSCLC patients will respond to anti-PD-1/PD-L1 antibody treatment. However, the majority of patients have no or only transient effect of the immunotherapy. There is therefore an unmet medical need for further refinement of the NSCLC stage III and IV treatment. The clinical efficacy of PD-1/PD-L1 targeted therapy depends on the presence of a spontaneous T cell response against the cancer cells in the patient, which can be unleashed by the treatment. The lack of clinical responses to PD-1/PD-L1 antibody treatment is due to several mechanisms, with lack of an adequate spontaneous immune response against tumor antigens playing a major role. Recently, functionality of systemic CD4 T-cell immunity was reported to define clinical outcomes and susceptibility to PD-L1/PD-1 blockade (3).

Human telomerase reverse transcriptase (hTERT) is a tumor-associated antigen over-expressed in more than 85% of solid tumors (4–6). hTERT expression is also one of the hallmarks in cancer as hTERT is required for tumor immortality and it is therefore an important target for anticancer therapy. Over the the past 15 years, several trials with hTERT-based vaccines have been conducted (7, 8). Telomerase expression is absent in most normal tissues, but present at low levels in hematopoietic stem cells, epithelial cells in colonic crypts and human germline cells (9, 10). Previous clinical trials showed no toxicity to these tissues after vaccination. In addition, normal bone marrow function was verified (11, 12).

UV1 is a therapeutic cancer vaccine consisting of three synthetic long peptides covering an epitope rich sequence within the active catalytic site of hTERT. The UV1 peptides were selected based on immunological analyses of blood from long-term cancer survivors previously treated with an unrelated first generation hTERT vaccine (GV1001) (13). All patients clinically benefitting from the vaccination demonstrated very broad CD4+ T-cell responses against selected hTERT peptides unrelated to the vaccine peptide given. The hTERT peptides most frequently recognised in GV1001 vaccinated patients with epitope spreading now make up the UV1 vaccine. Furthermore, these analyses have shown that UV1 peptides are promiscuous with respect to HLA class II molecules, ensuring a broad population coverage without the need to HLA-type patients as part of the inclusion criteria (13, 14). Immunization with UV1 peptides predominantly aims to induce Th1 immune responses with secretion of interferon gamma (IFN-γ), tumor necrosis factor alpha (TNFα), and interleukin-2 (IL-2) to stimulate expansion of effector cells, such as cytotoxic CD8+ T cells, and activate other players of the immune system [recently reviewed in Borst et al. (15)].

UV1 is administered with granulocyte-macrophage colony-stimulating factor (GM-CSF) as an adjuvant and has previously been investigated in clinical phase I trials involving metastatic hormone-sensitive prostate cancer (14) and malignant melanoma in which UV1 was given in combination with ipilimumab (NCT02275416).

The current paper reports findings from a phase I clinical trial of repeated UV1 vaccine treatments in patients with locally advanced or metastatic NSCLC. Three different doses of UV1 were explored.

## Material and Methods

### Study Design

This study was an open-label, single-center, dose-finding phase I clinical trial, with the primary objective to investigate the safety and tolerability of the hTERT peptide therapeutic cancer vaccine UV1. Other primary endpoints were determination of immune responses against the vaccine and its peptide components. Secondary endpoints were tumor response [radiologically assessed by computed tomography (CT) scans] and progression-free survival (PFS). PFS was defined as time from date of treatment initiation to objective tumor progression or death. The study was amended in order to follow immune response for 5 years and overall survival (OS) for 10 years, allowing determination of long-term immune responses and safety. The clinical trial UV1/hTERT-L was performed with approval from the institutional protocol board, the Regional Committees for Medical Research Ethics - South East Norway (2012/1114, EudraCT 2012- 001852-20) and The Norwegian Medicines Agency approval and is registered on Clinicaltrials.gov (NCT01789099). All patients provided written informed consent to participate and the trial was conducted in accordance with Good Clinical Practice (GCP) and with the World Medical Association Declaration of Helsinki. Enrollment started in April 2013 and was terminated in June 2015. Survival analyses were censored in September 2019. No formal statistical hypothesis testing was planned, as this was a phase I study with limited number of patients.

### Patient Inclusion

The study enrolled patients with stage III or IV NSCLC treated with palliative radiotherapy and/or at least one line of platinum-based, doublet chemotherapy, who had stable disease or better confirmed by CT at least 4 weeks after last treatment. They had an Eastern Cooperative Oncology Group (ECOG) performance status ≤2 and adequate renal, hepatic, and hematological function. Patients with brain metastasis were excluded.

### Therapeutic Cancer Vaccine UV1 and Treatment Schedule

The UV1 vaccine (Ultimovacs ASA) consists of three synthetic hTERT peptides; one 30-mer and two 15-mers in equimolar ratio (14). UV1 was reconstituted in water for injection before administration by intradermal injection to the patient. Three different UV1 doses were investigated; 100, 300, and 700 µg. GM-CSF (75 µg) (Leukine^®^, Sanofi Aventis) was used as adjuvant. Both products were administrated as an intradermal abdominal injection, GM-CSF 10-15 minutes prior to UV1, at the same injection site.

The three first patients were treated with 100 µg UV1, the next three with 300 µg UV1 and the three following patients were treated with 700 µg UV1. Thereafter, one patient on each dose level was enrolled, starting with the lowest dose until a total of 18 patients were enrolled. The treatment was three times during the first week (days 1, 3, and 5) and then week 2, 3, 4, 6, 8, 10, 14, 18, 22, and 26 with the rationale of mimicking an acute infection/inflammation the first week followed by booster vaccinations. After week 26, additional vaccinations every 3 months up to 4 years were allowed, provided acceptable safety.

### Safety Evaluation

Safety evaluation included medical history, records of vital signs, physical examination and blood sampling at each treatment visit and 30 days after administration of the last dose of UV1. Adverse events (AEs) were assessed according to the National Cancer Institute Common Terminology Criteria for Adverse Events (CTCAE) version 4.0.

### Assessment of Clinical Response

Tumor response was evaluated using RECIST 1.1 based on CT scans of thorax and abdomen before entering the trial and every 3 months during the treatment period.

### Immunologic Assessment of UV1-Specific T-Cell Response

Peripheral blood mononuclear cells (PBMCs) were obtained from 50 ml peripheral blood taken in acid citrate dextrose (ACD) tubes prior to the first UV1 vaccination, after 2 weeks, 6 weeks, 10 weeks, and then, every 4 weeks including week 26. PBMCs were thereafter sampled every 3 months. The PBMCs were isolated and frozen as previously described (14). The UV1-specific proliferative response was determined using thawed PBMCs from each time point which were pre-stimulated and tested as previously described (14). The cells were seeded in 24-well plates at 2 × 10^6^ cells/well in CellGro DC medium (CellGenix GmbH, Freiburg, Germany). The PBMCs were stimulated with UV1 vaccine peptides (peptide 725; hTERT 691-705 (RTFVLRVRAQDPPPE), peptide 719-20; hTERT 660-689 (ALFSVLNYERARRPGLLGASVLGLDDIHRA), peptide 728; hTERT 651-665 (AERLTSRVKALFSVL) (Bachem AG, Bubendorf, Switzerland) at a concentration of 10 µM for each peptide. On day 3 of culture, IL-2 and IL-7 at concentrations of 20 U/ml and 5 ng/ml, respectively, were added. Cells were split or medium added if required during culture for 10–12 days. Responder T cells were re-stimulated with peptide-loaded autologous irradiated antigen presenting cells (APCs), and proliferation was determined by ^3^H-Thymidine incorporation assay as previously described, with all conditions tested in triplicates. The stimulation index (SI) was calculated by taking mean counts of wells containing T cells and irradiated APCs loaded with UV1 peptide divided by mean counts of control wells containing T cells and irradiated APCs without peptide. SI ≥ 3 was considered a positive UV1-specific T cell response. Patients with a spontaneous UV1-specific T-cell response in baseline samples (SI ≥ 3) were considered as immune responders if their response (SI) was increased 2-fold or more after UV1 vaccination for any of the three UV1 peptides compared to baseline in at least one post vaccination sample and/or as an increase in the number of peptides recognized.

### IFN-γ ELISPOT

IFN-γ ELISPOT assays were performed essentially as previously described with pre-stimulated PBMCs from the pre-stimulated cultures above, if cell numbers were sufficient (14). Responder T cells harvested on days 10–12 were seeded in triplicates in complete culture medium without serum at 0.1 × 10^6^ T cells/well and stimulated with autologous PBMCs at an effector: target (E:T) ratio of 2:1. UV1 peptides were added at 10 µM for each peptide and negative controls with T cells and APCs and T cells only and with SEC-3 superantigen (Toxin Technology Inc. Sarasota, FL, USA) (0.1 μg/ml) stimulated positive controls. Plates were incubated for 16–20 h prior to the addition of detection reagents and substrate. Spots were enumerated using an automated analyzer, CTL IMMUNOSPOT S5 VERSA-02-9030 (Cellular Technology Ltd, Shaker Heights, OH, USA). Specific spots were calculated by subtracting the mean number of spots + (2×SD) of the medium-only control from the mean number of spots of experimental wells.

### Intracellular Cytokine Staining Flow Cytometry

UV1-stimulated T cells were thawed and resuspended in X-vivo 15 medium (Lonza) before co-culture with either autologous Epstein Barr virus transformed B-cell lines (EBV-LCLs) or autologous PBMCs loaded with CellTrace Violet (Life Technologies Carlsbad, CA, USA) for gating purposes and loaded or not with the UV1 mix of peptides (10µM). E:T ratio was 1:2. Cells were co-cultured for 10 h in the presence of GolgiStop and GolgiPlug (both BD Biosciences, San Jose, CA, USA) at a dilution of 1/1000. After 10 h, cells were harvested, washed in staining buffer consisting of phosphate buffered saline (PBS) containing 2% Fetal Calf serum (FCS) before staining using the PerFix-nc kit (Beckman Coulter, Brea CA, USA) following the manufacturer’s instructions. The following antibodies were used: anti-CD4-PECy7 (clone RPA-T4), CD8-BV-605 (clone RPA-T8, BioLegend, San Diego CA, USA), CD3-APC (clone OKT3) TNF-α-PE (clone MAb11, BD Biosciences), and IFN-γ (clone 4S.B3) All antibodies were purchased from eBioscience, except where otherwise noted. Cells were acquired on a FacsCanto flow cytometer and the data were analyzed using FlowJo software (Treestar Inc. Ashland, OR, USA). Cells were gated on lymphocytes in forward scatter (FSC) vs. side scatter (SSC), single cells, CellTrace negative cells, then CD4+CD3+ cells or CD8+CD3+ cells.

## Results

### Patient Characteristics and Treatment

Eighteen patients were enrolled between 2013 and 2015. The median age of patients was 65.2 years (range, 48–76); ten patients were female. All patients had stage III or IV NSCLC disease; eight patients had squamous cell carcinoma while ten had adenocarcinoma. The majority of patients (77.8%) had an ECOG performance status equal 0. Eight patients had received one prior line of platinum-based doublet chemotherapy, eight patients had received two lines and one patient had received four prior lines of chemotherapy. One patient had been treated with pembrolizumab before being enrolled in the study. Radiotherapy had been given to eight of the patients prior to enrollment. All patients had stable disease (SD) or better at least 4 weeks prior to inclusion in the study (**Table 1**).

**Table 1:** Baseline demographics.

UV1 dose (µg) Number of patients	100 6	300 6	700 6	All 18
**Age (year)**				
Mean (range)	65.4 (49–75)	63.5 (48–76)	66.8 (61–72)	65.2 (48–76)
**Sex**				
Female n (%)	2 (33.3)	5 (83.3)	3 (50.0)	10 (55.6)
Male n (%)	4 (66.7)	1(16.7)	3 (50.0)	8 (44.4)
**ECOG PS**				
0 n (%)	4 (66.7)	5 (83.3)	5 (83.3)	14 (77.8)
1 n (%)	2 (33.3)	1 (16.7)	1 (16.7)	4 (22.2)
**Stage**				
III n (%)	1 (16.7)	2 (33.3)	3 (50.0)	6 (33.3)
IV n (%)	2 (33.3)	3 (50.0)	2 (33.3)	7 (38.9)
III/IV n (%)	3 (50.0)	1 (16.7)	1 (16.7)	5 (27.8)
**Time since diagnosis (months)**				
Mean (SD)	21 (26)	29 (32)	16 (13)	22 (24)
Median (range)	10 (6; 72)	16 (7; 89)	12 (6; 39)	13 (6; 89)
**Histology**				
Squamous cell carcinoma	3 (50.0)	3 (50.0)	2 (33.3)	8 (44.4)
Adenocarcinoma	3 (50.0)	3 (50.0)	4 (66.7)	10 (55.6)
**Prior chemotherapy**				
No n (%)	0 (0.0)	1 (16.7)*	0 (0.0)	1 (5.6)*
Yes n (%)	6 (100.0)	5 (83.3)	6 (100.0)	17 (94.4)
**Prior radiotherapy**				
No n (%)	3 (50.0)	1 (16.7)	2 (33.3)	6 (33.3)
Yes n (%)	3 (50.0)	5 (83.3)	4 (66.7)	12 (66.7)

*Prior treatment with pembrolizumab.

The above characteristics were well distributed between the dosage groups. The mean time from primary diagnosis was 22 ± 24 months, the shortest was in the 700 µg dose group (16 ± 13 months), and longest in the 300 µg dose group (29 ± 32 months). The median time from diagnoses across all three dosage groups was 13 months (range, 6–89). The treatment schedule is outlined in **Supplementary Figure 1**. Treatment with UV1 was put on hold in October 2015 due to safety concerns (serious allergic reactions) in other clinical studies with UV1. Thereafter no further vaccinations were given to any participants in this study. The maximum number of UV1 and GM-CSF doses given was 18 (range, 9–18) for UV1 and 18 (range, 2–18) for GM-CSF.

### Safety

The treatment was generally safe and well tolerated. Overall, all patients experienced one or more adverse events (AEs), the majority (55.6%) of mild intensity (grade 1). Adverse events were mainly injection site reactions. Three AEs grade 3, all not related to treatment, while no grade 4 were reported. No related serious adverse events or any dose limiting toxicities were observed. Most of the AEs were observed for the 300 µg dose group (46 events), closely followed by the 100 µg dose group (41 events). The number of events in the 700 µg dose group was lower (30 events). One patient (300 µg UV1) experienced two mild hypersensitivity reactions (grade 1) during the first week of treatment and GM-CSF was subsequently stopped after two treatments. UV1 was thereafter given alone for the rest of the treatment period.

One patient (700 µg UV1) was hospitalised with cholecystitis (infective) and cholelithiasis (grade 3). There was no causal relationship with the study medication in either of these two events. The patient recovered without sequela. The two other grade 3 AEs were pain in shoulder and worsening of arthritic pain. None of the events were related to study medication and both occurred in the lowest dose group (100 µg UV1). The number of patients with treatment-related AEs is presented in **Table 2**. The number and percentage of patients with AEs by preferred term according to frequency is presented in **supplementary Table 1**.

**Table 2:** Number of patients with one or more treatment-related AEs.

UV1 dose (µg) Number of patients	100 6	300 6	700 6	All 18
Injection site erythema	2	0	3	5
Fatigue	0	1	3	4
Injection site pruritus	2	0	2	4
Injection site reaction	2	1	0	3
Influenza like illness	1	1	0	2
Arthralgia	0	0	1	1
Blood pressure decreased	0	1	0	1
Erythema	0	1	0	1
Headache	0	1	0	1
Hypersensitivity	0	1	0	1
Injection site hyperaesthesia	1	0	0	1
Injection site pain	1	0	0	1
Injection site rash	1	0	0	1
Musculoskeletal pain	0	1	0	1
Pruritus	0	1	0	1
Tongue movement disturbance	0	1	0	1

### Immune Response

**Figure 1:**
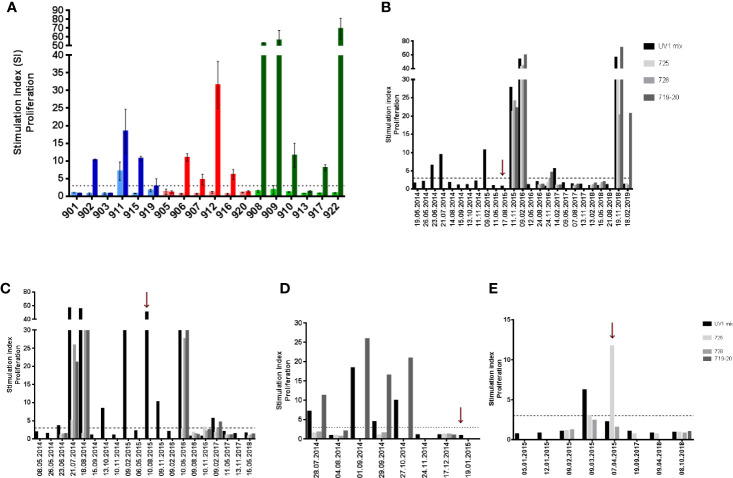
Summary of pre- and post-vaccination UV1-specific T-cell responses. T-cell proliferation against UV1 peptides in pre- and post-vaccination blood samples from the 18 patients evaluable for immune responses. The graph shows the strongest post-vaccination T-cell responses detected against the UV1 peptide mix for each patient **(A)**. Proliferation was measured in response to peptide-loaded PBMC by ^3^H-thymidine incorporation. A stimulation index (SI) of >3 is considered as an immune response. Stimulation index is plotted as mean of triplicates +/- standard deviation. Twelve of eighteen patients (12/18) had an immune response (67%). In blue: 100µg dose group, in red: 300µg dose group and in green: 700µg dose group (light: pre-vaccination, dark: post-vaccination). T-cell responses against the UV1 vaccine mix and its individual peptide components (725, 728, and 719-20) at all time points measured in patients 908, 700 µg dose **(B)**, 909, 700µg dose **(C)**, 911, 100µg dose **(D)**, and 916, 300µg dose **(E)**. Red arrows indicate when the last vaccine dose was given **(B–E)**.

All patients were evaluable for immune response monitoring. UV1-specific T cell responses were recorded in 12 patients (67%). One patient exhibited a spontaneous, pre-vaccine response against UV1. In the other eleven patients an immune response against the UV1 vaccine with a median stimulation index (SI) of 15.1 (4.7–69.1), well above the cut-off SI of 3 was demonstrated. These eleven patients developed *de novo* responses against UV1 peptides during the treatment period (**Figure 1A**). Representative examples of the longitudinal dynamics of the immune responses for four individual patients are given in **Figures 1B–E**. Patient 908 is of particular interest (**Figures 1B** and **3A, B** and **Supplementary Figure 2**). This patient developed an intermediate immune response against UV1 1 month after start of vaccination. During the next 17 months, the responding T cells were mostly undetectable in peripheral blood. Then, 18–21 months after start of treatment (3 months after the last vaccine dose), a very strong and broad immune response against UV1, caused by recognition of all three peptides, was detected. This response subsequently weaned off until the patient again demonstrated a very strong immune response against the same three peptides 2 years later, more than 3 years after end of treatment. This intriguing dynamics of the immune response most likely reflects a complex set of interactions between the immune system and the cancer in this patient. A similar pattern was observed when data from patient 909 were analyzed (**Figure 1C**). An intermediate T cell response against UV1 was detected 1.5 months after start of vaccination. The response developed into a broad and strong T cell response against all peptides 2–3 months after start of vaccination, then disappeared and reappeared during the 15-month vaccination period before a broad and strong T cell response was again detected around 9 months after end of treatment. Two years later, the response was no longer detectable. Furthermore, the data displayed in **Figures 1B–E** illustrate that concomitant tumor may profoundly influence the dynamics of the immune response. Patient 911 had a spontaneous UV1-specific T cell response at baseline (28.07.2014), which turned out to be directed against peptide 719-20 (**Figure 1D**). This response was boosted with UV1 vaccination, but no responses against the two other UV1 peptides were recorded. Data from patient 916 shown in **Figure 1E** demonstrate a T cell response against the 725 peptide. Patient 916 received only two doses of GM-CSF (due to grade 1 hypersensitivity) and was given the remaining UV1 doses (7 doses) without adjuvant, which may have resulted in a suboptimal vaccine response in patient 916. This patient had been treated with pembrolizumab before entering the UV1 study. In both patients 911 and 916, who received the two lower doses of UV1, the responses were of intermediate strength.

**Figure 2:**
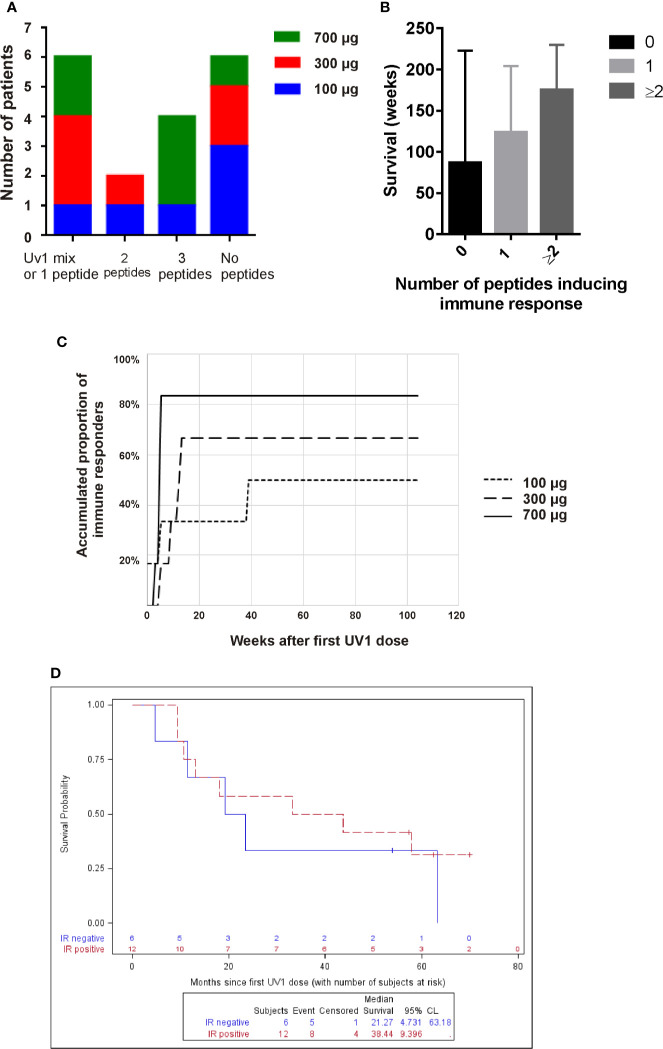
High UV1 dose improves immune response which correlates with improved OS. The number of patients responding to one (or only UV1 mix), two or three, or no peptides is shown in **(A)**. In **(B)**, the survival (weeks) is shown for patients responding to no peptides, one peptide or two or more. The time-to-response and accumulated proportion of immune responders per UV1 dose group is shown in **(C)**. Overall survival (OS) in immune responders versus non-immune responders is shown in **(D)**.

When summarizing the immune responses against individual peptides we observe that four patients responded to all three peptides of the UV1 vaccine (three patients received 700 µg, one 100 µg). Two patients responded to two peptides, while four to a single peptide only. Two patients (907, 912) were only tested against the UV1 mix and both were responders (**Figures 1A** and **2A**). The individual patterns of recognition indicate that, following processing of the UV1 vaccine, each patient selects epitopes that are able to be presented by the particular patient’s HLA class II molecules. It should be noted that immune monitoring was not set up to analyze HLA class I restricted T cell responses during the trial due to lack of information on HLA types and limited availability of material. Furthermore, a broad immune response correlated with improved survival; however, this was not significant (**Figure 2B**).

After 13 weeks on study, 50% of the patients showed a UV1-specific immune response (**Figure 2C**). In three patients, all in the lowest dose group, immune responses were detected at later time points. A higher proportion of patients had immune response in the 700 µg dose group (83%) compared with the 300 µg and 100 µg groups (67% and 50%, respectively), establishing a dose response relationship in this UV1 trial. Notably, the higher response rate in the 700 µg dose group could not be explained by the higher number of stage III patients in this dose group, since the immune response rate across all doses was 67% in stage III patients vs. 60% in stage III/IV and 71% in stage IV patients.

When looking at the OS versus detectable immune response or no immune response, the median OS in patients without immune response (N = 6) was 21.3 months compared to 38.4 months (p = 0.64) in the group of patients with immune response (N = 12), as illustrated in **Figure 2D**.

**Figure 3:**
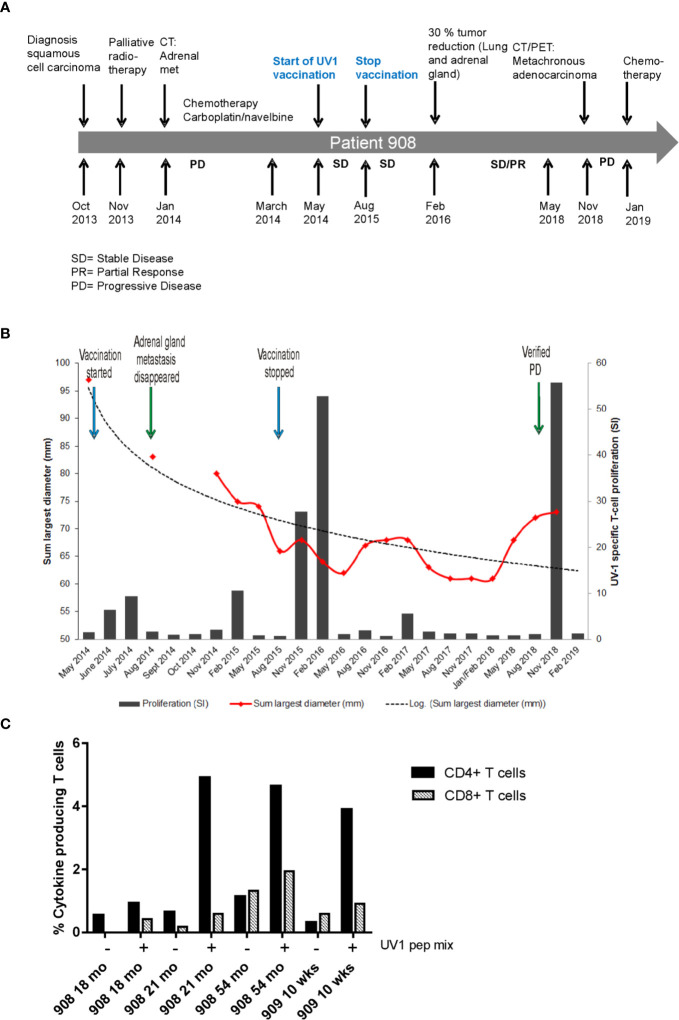
Tumor reduction and Th1 cytokine production post vaccination. Timeline for diagnosis, treatment and disease progression in patient 908 **(A)**. Summary of pre- and post-vaccination UV1-specific T-cell responses detected, and tumor reduction measured in patient 908 **(B)**. T-cell proliferation against UV1 peptides in blood samples pre- and post-vaccination at all time points tested from patient 908. Proliferation was measured in response to peptide-loaded PBMC by ^3^H-thymidine incorporation. A stimulation index (SI) of ≥3 is considered as an immune response. Dotted line is the logarithmic trend line for tumor diameter. Th1 cytokine production in T cells stimulated with UV1 peptides post vaccination is shown in **(C)**. TNF-α and IFN-γ production was measured by intracellular cytokine staining in PBMCs pre-stimulated with UV1 peptides. T cells from indicated time points from two patients were re-stimulated with autologous APCs, either PBMCs or B-cell lines (EBV-LCLs) loaded with UV1 peptides (10 µM) for 10 h. CD4+ T-cell response is indicated by black bars and CD8+ T-cell response by hatched bars.

Patient 908 was diagnosed with squamous cell carcinoma stage T4N1, stage IIIA in October 2013 (**Figure 3A**) and received palliative radiotherapy to a large tumor in the right lung prior to study inclusion. CT scan after radiotherapy demonstrated reduced tumor in the right lung, in addition to a new 22 mm metastasis in the left adrenal gland with necrosis. Four courses of carboplatin/navelbine based chemotherapy were then provided. At inclusion in the study (May 2014), the tumor in the right lung was reduced and the left adrenal metastasis was measured to be 14 x 17 mm. After 24 months the patient had achieved a more than 30% reduction in the size of the lung and adrenal tumors. The radiologist concluded with stable disease from May 2014 until May 2018 (48 months duration). Concomitant with the largest reduction of tumor size measured (May 2016, month 24), a strong immune response was recorded (**Figure 3B**). In contrast, no or only weak immune responses were recorded between May 2016 and November 2018 when a new, strong immune response was recorded, and new lesions were detected. A PET scan concluded with a tumor in the left lung, mediastinal glands and a tumor in the opposite lung. Endobronchial ultrasound (EBUS) was performed and a fine-needle aspiration cytology (FNAC) from left side concluded with adenocarcinoma and progressive disease (PD). Three months later, after the patient had started a second round of chemotherapy (January 2019) due to the PD, the immune response was reduced yet still positive against one of the vaccine peptides.

Very few patients had sufficient numbers of T cells for testing in IFN-γ ELISPOT after pre-stimulation with peptides *in vitro.* A few time points were tested and correlated with proliferative T-cell responses against UV1 peptides in the same patients (**Supplementary Figure 2**). However, for two of the patients, frozen, pre-stimulated T cells from time points with positive proliferative responses were thawed and stimulated with UV1 peptides loaded on autologous APCs (PBMC or EBV-LCL) for 10 h for intracellular cytokine staining. These patients showed clear cytokine production, mainly by CD4+ T cells (**Figure 3C** and **Supplementary Figure 3**) after re-stimulation. The main cytokine produced was TNF-α, and some IFN-γ demonstrating that the UV1 vaccine induces a Th1 type immune response in these patients.

### Tumor Response

Seventeen of the 18 patients had baseline CT and at least one post UV1 treatment CT and were evaluable for tumor response according to RECIST 1.1. Fifteen patients had stable disease (SD) throughout the study as best response. One patient, in the 700 µg UV1 dose group, had a reduction of more than 30% in the overall tumor burden/diameter between 15 and 24 months after start of UV1 treatment. However, the patient had radiation to the lung prior to the enrollment, and it was therefore difficult to determine whether this patient had achieved a real partial response induced by the vaccination or radiotherapy. This patient has been described in more detail above under the reporting of immune responses.

### Survival

**Figure 4:**
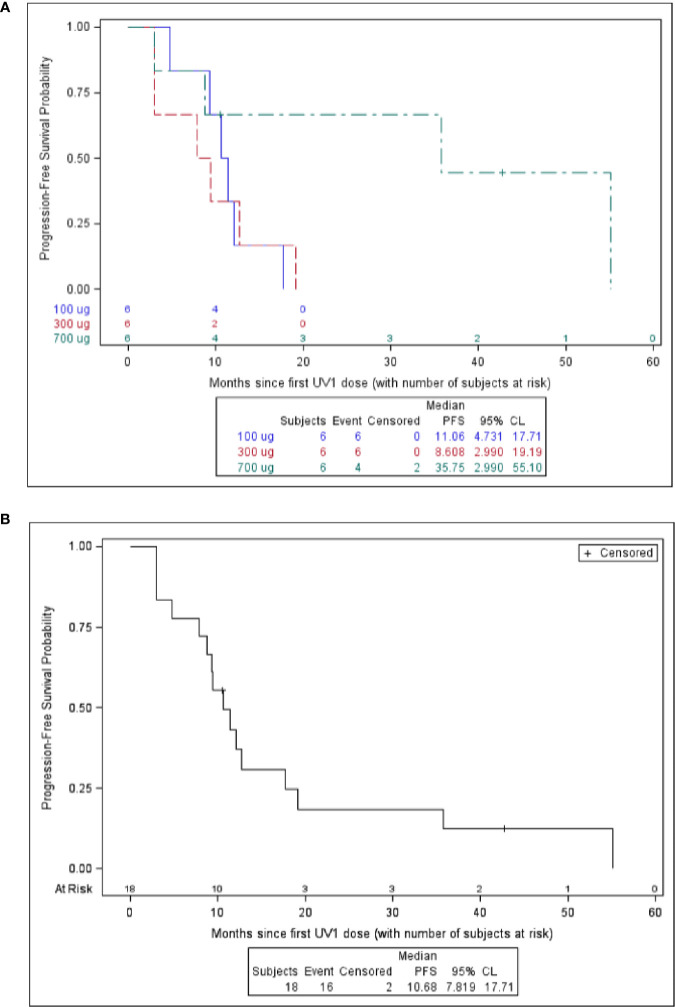
Survival versus months since first UV1 dose. Overall survival is shown in **(A)** and progression free survival is shown in **(B)**.

Overall survival (OS) at one year after first UV1 treatment was 72%. Overall survival at two, three, and four years was 50, 44, and 39%, respectively (**Table 3**). Three patients (50%) in the 700 µg dose group were diagnosed with stage III disease, while the corresponding numbers in the other dose groups were one (100 µg) and two (300 µg) patients (**Table 1**). Five patients were alive as of March 2020 (median survival 5.56 (4.84–5.91) years); four in the 700 µg dose group and one in the 300 µg dose group and none of these patients are known to have received anti-PD-1 therapy after UV1 treatment. Median OS across the dose groups was 28.2 months. **Figure 4A** shows Kaplan-Meier plot of OS for the patients in the three dose groups as of March 2020. A clear relationship between vaccine dose and OS was observed. Progression-free survival (PFS) was defined as the time from start of vaccination to the date of the first documented tumor progression or death due to any cause. Median PFS for all patients was 10.7 months, as shown in the Kaplan-Meier plot in **Figure 4B**.

**Table 3:** Overall survival per dose group and overall.

	Dose group			All patients
	100 µgN = 6	300 µgN = 6	700 µgN = 6	N = 18
One-year survival, n (%)	2 (33%)	5 (83%)	6 (100%)	13 (72%)
Two-year survival, n (%)	1 (17%)	3 (50%)	5 (83%)	9 (50%)
Three-year survival, n (%)	1 (17%)	2 (33%)	5 (83%)	8 (44%)
Four-year survival, n (%)	0	2 (33%)	5 (83%)	7 (39%)
Median survival, months	11.1	26.2	Not reached	28.2

N = no. of patients treated in the dose group.

n = number of patients alive.

% = percentage of patients in that dose group.

## Discussion

Activation of the hTERT gene is strongly implicated in the pathogenesis of NSCLC and a high level of hTERT expression is a negative prognostic factor in early disease (16). Furthermore, amplification of the hTERT gene is an independent marker of poor prognosis in NSCLC associated with short disease-free survival (17). Mutations in the hTERT promoter region are also a negative prognostic marker in NSCLC, although the frequency of such mutations is low (18). High levels of hTERT seem to result in spontaneous activation of hTERT-specific CD4+ T cells in metastatic NSCLC patients, as reported by Godét et al. (19), who observed a significant frequency of Th1 responses against several hTERT epitopes (38% of patients, n = 84) in pre-treatment blood samples. Such T cell responses were associated with better prognosis after primary chemotherapy. To our knowledge the role of these pre-treatment hTERT-specific T cells in checkpoint inhibitor therapy has not been investigated in NSCLC. We have previously observed spontaneous Th1 responses against the GV1001 epitope in advanced NSCLC patients after prior chemotherapy/radiotherapy (20), although at a much lower frequency. In the current UV1 study we observed a single patient with a spontaneous UV1-specific hTERT response, whereas other studies using a different panel of hTERT peptides have detected a higher level of spontaneous hTERT responses in therapy-naïve NSCLC patients (21). In late stage cancer patients hTERT-specific T cells become exhausted and can be found in a reduced proportion of patients which may explain our low baseline responses (21). The presence of spontaneous hTERT-specific T-cell responses boosted by vaccination and the lack of toxicity in vaccinated patients, show that despite the low level expression of telomerase in certain normal tissues such as hematopoietic stem cells and keratinocytes, vaccines targeting hTERT are safe and immunogenic. Together these data provide evidence for the strong immunogenicity of hTERT and its role as a legitimate target for cancer immunotherapy (6).

Treatment with UV1 was put on hold (October 2015) in the three UV1 phase I studies due to serious allergic reactions related to high levels of IgE against GM-CSF and UV1, mainly in the prostate cancer study (14). However, in the current study none of the patients experienced allergic reactions compatible with low levels of IgE against GM-CSF and UV1 (*unpublished data*). The exposure of GM-CSF and UV1 in the current study was comparable with respect to number of doses given at the different dose levels in the other studies.

UV1-specific T cell responses were recorded in 12/18 patients (67%); 83% in the 700 µg dose followed by 67 and 50% in 300 and 100 µg dose groups, respectively. The highest dose induced UV1-specific immune responses that were stronger and occurred earlier compared to the immune responses reported for the two lower doses and together with the safety data, provide strong support for future use of the 700 µg dose in this patient group. This optimal dose of UV1 vaccine is expected to induce a higher number of immune responses in future NSCLC studies than in the current dose-finding study. The immunogenicity of the vaccine in a broad, non-HLA selected population (**Supplementary Table 2**) is due to a high number of T cell epitopes present in the vaccine which allows each patient to tailor an immune response based on his/her own HLA molecules following processing in antigen presenting cells (13) (and *unpublished data*).

In the longitudinal immune monitoring studies, exemplified by patient 908, an intriguing fluctuation in UV1-specific T cell levels in peripheral blood of the patients was revealed. Interestingly, coinciding with a >30% reduction in tumor mass, a very strong immune response towards all peptides in the UV1 vaccine was reported at 18 months and was still present 3 months later. The patient subsequently remained stable for 3 years before progression, when a metachronous lung cancer (an adenocarcinoma) was reported. During this period the immune response was undetectable or low. Interestingly, with the progression, a new wave of a broad and strong T cell activity against UV1 appeared and was still detectable 3 months after, though reduced after start of chemotherapy. The patient received no other antitumor treatment during this 4.5 year period. A sudden strong immune response observed in peripheral blood is often correlated with tumor reduction (22). This may explain the first peak of strong UV1 immune responses and has previously been well described in similar hTERT peptide vaccination studies in NSCL (11, 20) and melanoma (23). Furthermore, the same type of expansions and contractions of tumor-specific T cells have been mechanistically described in mouse models of spontaneous breast cancer and fibrosarcoma demonstrating the importance of systemic immune responses (22, 24). One may speculate that the second peak of high T cell activity observed in peripheral blood in this patient almost 5 years after start of vaccination may have been triggered by stimulation of UV1-specific memory T cells by the new, metachronous cancer.

Survival data demonstrate a 12-month OS of 72%, 24-month of 50%, 44% at 36 months, and 39% at 48 months. Five years survival is not reached for the patients in the 700 µg dose group at data cut-off (March 2020). Five patients are alive (28%); four in the 700 µg dose group and one in the 300 µg dose group. The stage of the disease was favorable in the 700 µg dose group, with 50% stage III. None of the five patients alive (median survival, 5.56 years; range, 4.84–5.91) have been reported to have received further immunotherapy after termination of UV1 treatment. Two patients were treated with anti PD-1 antibody after progression in the UV1 study and died later, while one patient received pembrolizumab before entering the UV1 study and is alive. This patient was treated with chemo- and radiation therapy after disease progression in this study. The median time to death in the two lowest dosage groups was 11.1 months (4.7–43.8) and 26.2 months (9.4-NR), respectively. In the 700 µg dose group two patients have died, and this occurred 79 weeks and 251 weeks after first UV1 treatment. Although the number of patients in this study is low, the combined data from the three different dose cohorts all go in the same direction, pointing to a correlation between vaccine doses, number of immune responders, rapidity of the induction of immune responses, and clinical outcome. The patients without immune response had a median OS of 21.3 months, while the corresponding OS was 38.4 months in the patients with immune response indicating a potential effect of the immune response on survival.

Median PFS for all patients was 10.7 months, while the median OS across the dose groups was 28.2 months.

We have seen some encouraging results in previous studies with the first generation hTERT (GV1001) peptide vaccine in NSCLC patients (11, 20). In these single agent vaccine studies, immunological responses were also associated with prolonged survival. With the current understanding of the role of immune checkpoints in inhibiting spontaneous anti-tumor immune responses, and the clinical efficacy of anti-PD-1 antibody treatment in patients with NSCLC (25), it is tempting to speculate that the true potency of UV1 and other hTERT vaccines can only be explored in combinations with immune checkpoint inhibitors, where the role of the vaccine is to prime a meaningful T cell response and the role of the checkpoint inhibitors will be to enhance the response and to facilitate the activity of the T cells in the tumor microenvironment. Recent reports that emphasize the role of CD4+ T cells in determining the clinical outcome of immune checkpoint blockade in patients with NSCLC (3) and melanoma (26) are of particular relevance in the context of the UV1 vaccine, which is designed to elicit CD4+ T cell responses. Moreover, PD-1/PD-L1 inhibition in an orthotopic murine model of lung adenocarcinoma demonstrated that cancer cells expressing MHC II were sensitive to immune checkpoint inhibition whereas MHC II negative lung cancer was resistant (27). MHC II expressing tumors showed increased T-cell infiltration and Th1-derived cytokine production. Several pre-clinical studies have shown improved anti-tumor responses upon combination of vaccine and checkpoint inhibitors (28, 29). Data from clinical studies combining anti-immune checkpoint blockade and vaccines are still limited, but some early trials show encouraging results (30) and were recently reviewed (31). PD-1 and CTLA-4 checkpoints differentially affect CD8+ and CD4+ T-cell phenotypes (32) and recent data indicate that immune responses induced by anti-CTLA-4 and anti-PD-1 checkpoint-blockade are driven by different cellular mechanisms (33). This would indicate that combination studies of vaccination and immune checkpoint inhibitors should take into account the type of immune response preferentially induced by the vaccine before determining which checkpoint inhibitor to combine with as well as the timing and sequencing of the treatments.

### Conclusion

We have here documented the safety and immunogenicity of UV1 treatment in advanced NSCLC patients. The current study also provides preliminary evidence of clinical efficacy indicating improved OS in UV1 immune responders. hTERT is a universal tumor target and we have here demonstrated that UV1 provides a broad patient population coverage in its ability to generate an immune response after vaccination. Based on these data, a UV1 dose of 700 μg is optimal in this NSCLC population. The results encourage combination of the UV1 vaccine with immune checkpoint inhibitors to bring out the full potential of the vaccine and to enhance the clinical efficacy of immune checkpoint blockade in patients with NSCLC.

## Data AvaIlability Statement

The raw data supporting the conclusions of this article will be made available by the authors, without undue reservation.

## Ethics Statement

The studies involving human participants were reviewed and approved by Regional Committees for Medical Research Ethics - South East Norway. The patients/participants provided their written informed consent to participate in this study.

## Author Contributions

PB, EMI, GG, JK, and SA designed the study. PB, TG, and MN, included and treated patients, and evaluated clinical responses. CS-R performed radiology and evaluated tumor responses. HJ performed T-cell isolation, analyzed and interpreted T-cell responses. EMI designed experiments, performed flow cytometry, analyzed and interpreted immune responses, and wrote the manuscript. GG interpreted data and wrote the manuscript. PB and WR analyzed and interpreted the data and wrote the manuscript. All authors contributed to the article and approved the submitted version.

## Funding

This clinical trial has been financially supported by Ultimovacs ASA.

## Conflict of Interest

GG and EMI are inventors on the UV1 vaccine patent. WR, GG, and EMI are shareholders of Ultimovacs ASA. WR, GG, and SA are Ultimovacs ASA employees.

The remaining authors declare that the research was conducted in the absence of any commercial or financial relationships that could be construed as a potential conflict of interest.
