# Supplementary figures and images for: Long-Term Outcomes of a Phase I Study With UV1, a Second Generation Telomerase Based Vaccine, in Patients With Advanced Non-Small Cell Lung Cancer
Source: Front Immunol. 2020 Nov 26;11:572172. doi: 10.3389/fimmu.2020.572172 (PMC7726017; doi:10.3389/fimmu.2020.572172)

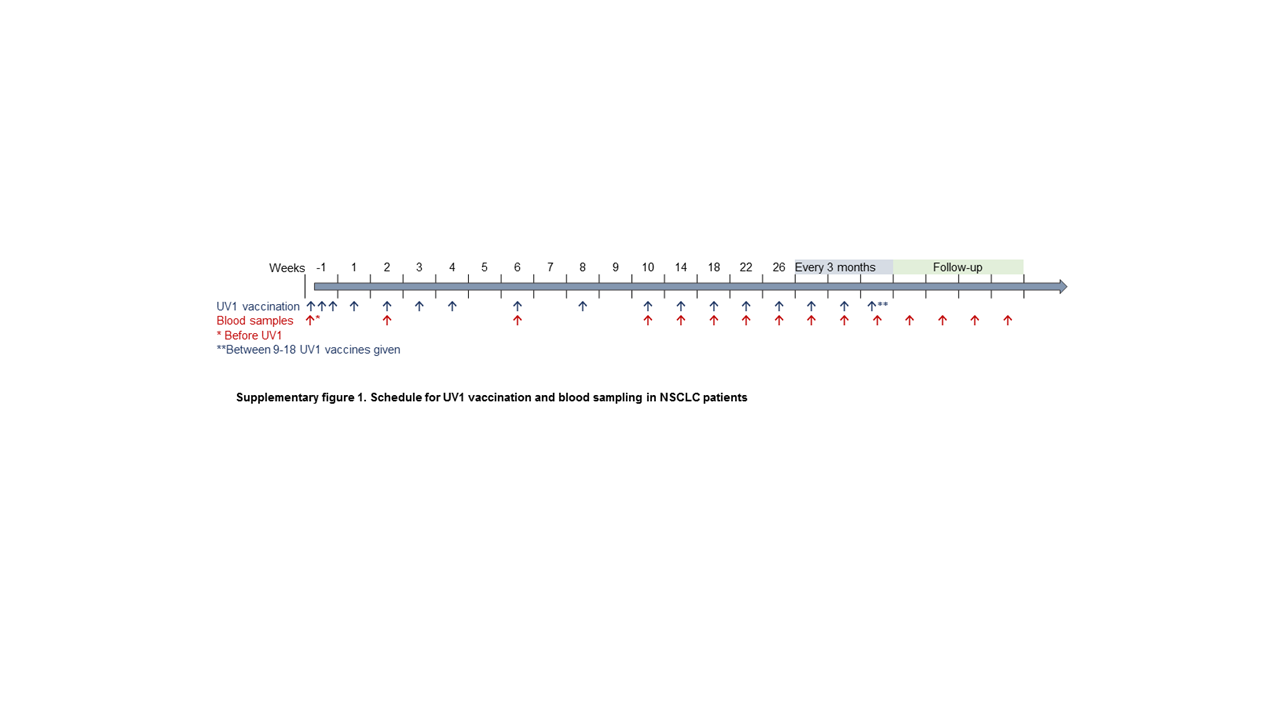

Supplement: Supplementary file 1 [file Image_1.tif]

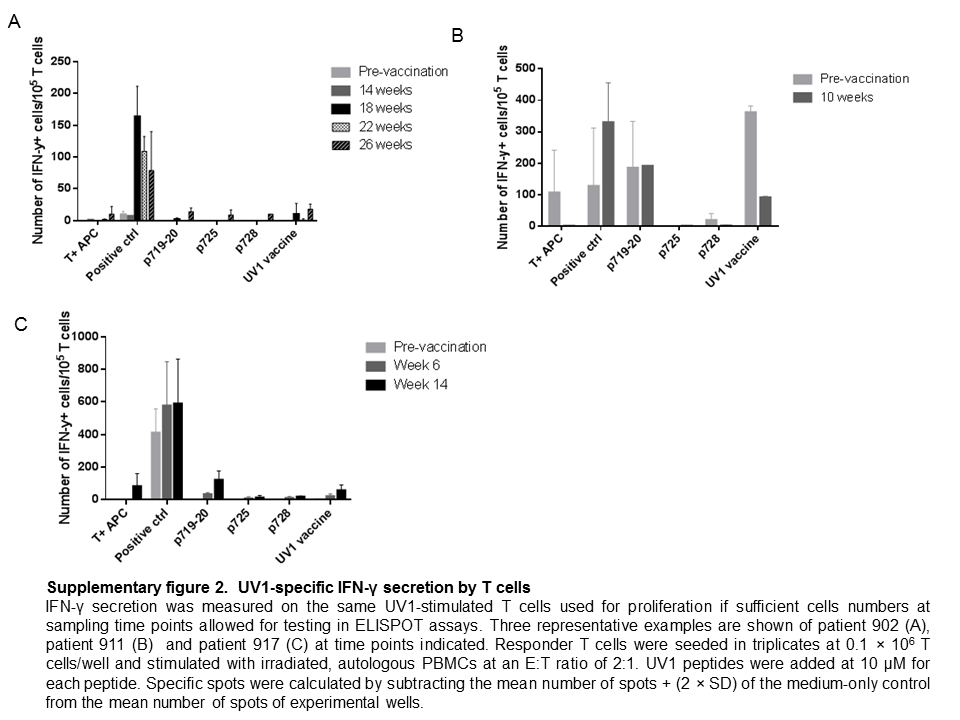

Supplement: Supplementary file 2 [file Image_2.tif]
