# Supplementary material for: Long-Term Outcomes of a Phase I Study With UV1, a Second Generation Telomerase Based Vaccine, in Patients With Advanced Non-Small Cell Lung Cancer
Source: Front Immunol. 2020 Nov 26;11:572172. doi: 10.3389/fimmu.2020.572172 (PMC7726017; doi:10.3389/fimmu.2020.572172)
Supplement: Supplementary file 4 [file Table_1.docx]

| **Supplementary Table 1: No and % (of N) of patients with AEs by preferred term according to frequency** | | | | | | | |  |  |
| --- | --- | --- | --- | --- | --- | --- | --- | --- | --- |
| Preferred term | Dose group | | | | | | All patients  N = 18 | | |
|  | 100 µg  N = 6 | | 300 µg N = 6 | | 700 µg N = 6 | |  |  |  |
|  | n | % | n | % | n | % | n | | % |
| Fatigue | 2 | 33.3 | 3 | 50.0 | 4 | 66.7 | 9 | | 50.0 |
| Nasopharyngitis | 1 | 16.7 | 2 | 33.3 | 3 | 50.0 | 6 | | 33.3 |
| Injection site erythema | 2 | 33.3 | 0 | 0 | 3 | 50.0 | 5 | | 27.8 |
| Breath sounds abnormal | 1 | 16.7 | 1 | 16.7 | 2 | 33.3 | 4 | | 22.2 |
| Dizziness | 1 | 16.7 | 3 | 50.0 | 0 | 0 | 4 | | 22.2 |
| Injection site pruritus | 2 | 33.3 | 0 | 0 | 2 | 33.3 | 4 | | 22.2 |
| Cough | 2 | 33.3 | 0 | 0 | 1 | 16.7 | 3 | | 16.7 |
| Dyspnoea | 3 | 50.0 | 0 | 0 | 0 | 0 | 3 | | 16.7 |
| Influenza like illness | 1 | 16.7 | 2 | 33.3 | 0 | 0 | 3 | | 16.7 |
| Injection site reaction | 2 | 33.3 | 1 | 16.7 | 0 | 0 | 3 | | 16.7 |
| Vomiting | 1 | 16.7 | 1 | 16.7 | 1 | 16.7 | 3 | | 16.7 |
| Arthralgia | 1 | 16.7 | 0 | 0 | 1 | 16.7 | 2 | | 11.1 |
| Back pain | 0 | 0 | 2 | 33.3 | 0 | 0 | 2 | | 11.1 |
| Chest pain | 0 | 0 | 2 | 33.3 | 0 | 0 | 2 | | 11.1 |
| Decreased appetite | 1 | 16.7 | 1 | 16.7 | 0 | 0 | 2 | | 11.1 |
| Depression | 0 | 0 | 1 | 16.7 | 1 | 16.7 | 2 | | 11.1 |
| Musculoskeletal pain | 1 | 16.7 | 1 | 16.7 | 0 | 0 | 2 | | 11.1 |
| Abdominal pain upper | 0 | 0 | 1 | 16.7 | 0 | 0 | 1 | | 5.6 |
| Blood pressure decreased | 0 | 0 | 1 | 16.7 | 0 | 0 | 1 | | 5.6 |
| Bradycardia | 0 | 0 | 0 | 0 | 1 | 16.7 | 1 | | 5.6 |
| Bronchitis | 1 | 16.7 | 0 | 0 | 0 | 0 | 1 | | 5.6 |
| Chest discomfort | 0 | 0 | 1 | 16.7 | 0 | 0 | 1 | | 5.6 |
| Cholecystitis infective | 0 | 0 | 0 | 0 | 1 | 16.7 | 1 | | 5.6 |
| Cholelithiasis | 0 | 0 | 0 | 0 | 1 | 16.7 | 1 | | 5.6 |
| Constipation | 0 | 0 | 1 | 16.7 | 0 | 0 | 1 | | 5.6 |
| Diarrhoea | 0 | 0 | 1 | 16.7 | 0 | 0 | 1 | | 5.6 |
| Dizziness postural | 0 | 0 | 0 | 0 | 1 | 16.7 | 1 | | 5.6 |
| Dyspepsia | 0 | 0 | 0 | 0 | 1 | 16.7 | 1 | | 5.6 |
| Dysphagia | 1 | 16.7 | 0 | 0 | 0 | 0 | 1 | | 5.6 |
| Erythema | 0 | 0 | 1 | 16.7 | 0 | 0 | 1 | | 5.6 |
| Gastrooesophageal reflux disease | 0 | 0 | 1 | 16.7 | 0 | 0 | 1 | | 5.6 |
| Haemoptysis | 0 | 0 | 0 | 0 | 1 | 16.7 | 1 | | 5.6 |
| Headache | 0 | 0 | 1 | 16.7 | 0 | 0 | 1 | | 5.6 |
| Hypersensitivity | 0 | 0 | 1 | 16.7 | 0 | 0 | 1 | | 5.6 |
| Injection site hyperaesthesia | 1 | 16.7 | 0 | 0 | 0 | 0 | 1 | | 5.6 |
| Injection site pain | 1 | 16.7 | 0 | 0 | 0 | 0 | 1 | | 5.6 |
| Injection site rash | 1 | 16.7 | 0 | 0 | 0 | 0 | 1 | | 5.6 |
| Joint swelling | 0 | 0 | 0 | 0 | 1 | 16.7 | 1 | | 5.6 |
| Lymph node pain | 0 | 0 | 1 | 16.7 | 0 | 0 | 1 | | 5.6 |
| Malaise | 0 | 0 | 1 | 16.7 | 0 | 0 | 1 | | 5.6 |
| Musculoskeletal chest pain | 0 | 0 | 0 | 0 | 1 | 16.7 | 1 | | 5.6 |
| Nausea | 0 | 0 | 1 | 16.7 | 0 | 0 | 1 | | 5.6 |
| Nightmare | 1 | 16.7 | 0 | 0 | 0 | 0 | 1 | | 5.6 |
| Pneumonia | 0 | 0 | 0 | 0 | 1 | 16.7 | 1 | | 5.6 |
| Pruritus | 0 | 0 | 1 | 16.7 | 0 | 0 | 1 | | 5.6 |
| Pyrexia | 1 | 16.7 | 0 | 0 | 0 | 0 | 1 | | 5.6 |
| Tinnitus | 0 | 0 | 1 | 16.7 | 0 | 0 | 1 | | 5.6 |
| Tongue movement disturbance | 0 | 0 | 1 | 16.7 | 0 | 0 | 1 | | 5.6 |
| Viral infection | 0 | 0 | 0 | 0 | 1 | 16.7 | 1 | | 5.6 |
| Vitamin B12 deficiency | 0 | 0 | 1 | 16.7 | 0 | 0 | 1 | | 5.6 |
| Weight decreased | 0 | 0 | 1 | 16.7 | 0 | 0 | 1 | | 5.6 |
| Ref. Table 14.3.1.1.2 N is the number of patients in each dose group. n is the number of patients with one or more AEs. | | | | | | | | | |
