# Supplementary material for: Long-Term Outcomes of a Phase I Study With UV1, a Second Generation Telomerase Based Vaccine, in Patients With Advanced Non-Small Cell Lung Cancer
Source: Front Immunol. 2020 Nov 26;11:572172. doi: 10.3389/fimmu.2020.572172 (PMC7726017; doi:10.3389/fimmu.2020.572172)
Supplement: Supplementary file 5 [file Table_2.docx]

| Supplementary Table 2: Patient HLA typing in evaluable patients. | | | | | | | | | | | |
| --- | --- | --- | --- | --- | --- | --- | --- | --- | --- | --- | --- |
|  | HLA-A class I | |  | |  | HLA-A class II | | | |  | |
| HLA-A | | HLA-B | | | HLA-DRB1 | | HLA-DQB1 | | HLA-DPB1 | | |
|  | |  | | |  | |  | |  | | |
| *01:01 | - | *08:01 | | - | *03:01 | *- | *02:01 | *- | *01:01 | | *04:01 |
| *02:01 | *03:01 | *15:01 | | *40:01 | *04:01 | *04:04 | *03:02 | - | *02:01 | | *04:01 |
| *03:01 | *24:02 | *07:02 | | *18:01 | *15:01 | *- | *06:02 | *- | *04:01 | | *04:02 |
| *03:01 | *26:01 | *07:02 | | *37:01 | *13:01 | *15:01 | *06:02 | *06:03 | *04:01 | | - |
| *02:01 | - | *15:01 | | *27:05 | *04:01 | *08:01 | *03:02 | *04:02 | *02:01 | | *04:01 |
| *01:01 | *32:01 | *37:01 | | *50:01 | *04:01 | *13:03 | *03:01 | *03:02 | *03:01 | | *04:01 |
| *03:01 | - | *07:02 | | - | *15:01 | - | *06:02 | - | *04:01 | | *04:02 |
| *01:01 | *24:02 | *40:01 | | *44:02 | *04:04 | *15:01 | *03:02 | *06:02 | *02:01P | | *04:01 |
| *02:01 | - | *15:01 | | *40:01 | *04:01 | - | *03:02 | - | *02:01 | | *04:02P |
| *01:01 | *02:01 | *08:01 | | *15:01 | *03:01 | *08:01 | *02:01 | *04:02 | *02:01 | | *04:01 |
| *02:01 | - | *08:01 | | *40:01 | *03:01 | *04:04 | *02:01 | *03:02 | *01:01 | | *02:01P |
| *01:01 | *24:02 | *07:02 | | *51:01 | *13:01 | *15:01 | *06:02 | *06:03 | *04:01P | | *20:01P |
| *02:01 | *68:01 | *07:02 | | - | *15:01 | - | *06:02 | - | *01:01 | | *05:01 |
| *11:01 | *26:01 | *27:05 | | *35:01 | *01:01 | *14:01 | *05:01 | *05:03 | *04:02 | | *16:01 |
| *01:01 | *02:01 | *08:01 | | *44:02 | *03:01 | *04:01 | *02:01 | *03:01 | *03:01P | | *04:02P |
| *02:01 | *26:01 | *07:02 | | *44:02 | *01:01 | *04:01 | *03:01 | *05:01 | *02:01 | | *04:01 |
| *02:01 | - | *13:02 | | *18:01 | *07:01 | *09:01 | *02:02 | *03:03 | *04:01 | | - |

Supplementary Table 2. Patient HLA typing in evaluable patients. HLA typing was conducted using Tier 1 Typing by PCR-sequence specific oligonucleotides (PCR-SSOP) to resolve major allele groups to 4 digits, with some degeneracy. Immune responses were observed across HLA subtypes.
